# Supplementary material for: Non-invasive and noise-robust light focusing using confocal wavefront shaping
Source: Nat Commun. 2024 Jul 2;15:5575. doi: 10.1038/s41467-024-49697-w (PMC11219997; doi:10.1038/s41467-024-49697-w)
Supplement: Supplementary file 1 — Supplementary Information [file 41467_2024_49697_MOESM1_ESM.pdf]

Non-invasive and noise-robust light focusing using confocal wavefront shaping
Supplementary material

Dror Aizik, Anat Levin

Department of Electrical and Computer Engineering, Technion, Haifa, Israel

Corresponding author: droraizik@campus.technion.ac.il

March 12, 2024

Abstract

This manuscript provides supplement information to "Non-invasive and noise-robust light focusing using confocal wavefront shaping".

1 Mathematical derivation

We provide a derivation of an image formation model using a transmission matrix formulation, and use it to prove the non-linear properties of our confocal score. This derivation explains why the confocal score can favor modulations that focus at a single spot despite the fact that it collects linear fluorescence feedback.

1.1 Image formation model

Consider a set of K fluorescent particles inside a sample, and denote their positions by $o_1, \ldots, o_K$. We assume the SLM in the illumination arm is illuminated with a spatially uniform plane wave and use the SLM to display a complex 2D electric field that we denote by $\mathbf{u}$. Although $\mathbf{u}$ is a 2D field, we reshape it as a 1D vector. We also use $\boldsymbol{\nu}$ to denote a $K \times 1$ vector of the field propagating through the sample at each of the K fluorescent sources.

The relation between $\mathbf{u}$ and $\boldsymbol{\nu}$ is linear and can be described as a multiplication by a (very large) matrix

$$\boldsymbol{\nu} = \mathbf{T}^{\mathbf{i}}\mathbf{u}, \tag{1}$$

where $\mathbf{T}^{\mathbf{i}}$ is the incoming transmission matrix, describing the forward coherent light propagation in the tissue. We note that $\mathbf{T}^{\mathbf{i}}$ is specific to the tissue sample being tested, and different tissue samples are described by very different transmission matrices. For thick tissue, $\mathbf{T}^{\mathbf{i}}$ can be an arbitrarily complex matrix incorporating multiple scattering events in the tissue. Likewise, if the light returning from the target is coherent, its propagation to the SLM of the imaging arm can be described as $\mathbf{T}^{\mathbf{o}}\boldsymbol{\nu}$, where $\mathbf{T}^{\mathbf{o}}$ is the coherent back-propagation transmission matrix.

We denote by $\boldsymbol{\zeta}$ the wavefront placed on the SLM of the imaging arm, and by $\boldsymbol{\mathcal{D}}(\boldsymbol{\zeta})$ a diagonal matrix with $\boldsymbol{\zeta}$ on its diagonal. Our SLMs are placed at the Fourier planes of the imaging system, and we denote by $\boldsymbol{\mathcal{F}}$ the Fourier transform of the wavefront from the SLM to the camera sensor. With this notation, coherent light propagating from the target particles to the sensor, through the SLM, can be expressed as

$$\boldsymbol{\mathcal{F}}\boldsymbol{\mathcal{D}}(\boldsymbol{\zeta})\mathbf{T}^{\mathbf{o}}\boldsymbol{\nu}. \tag{2}$$

In the fluorescent case, the emissions from different points are incoherent, hence the recorded intensity is the sum of emitted intensity from each fluorescent bead:

$$\mathbf{I} \propto \sum_k |\boldsymbol{\mathcal{F}}\boldsymbol{\mathcal{D}}(\boldsymbol{\zeta})\mathbf{T}^{\mathbf{o}}_{\downarrow,k}|^2 |\boldsymbol{\nu}_k|^{2\alpha}, \tag{3}$$

where $|\boldsymbol{\nu}_k|^2$ is the energy of the excitation light arriving at particle o_k, and $\mathbf{T}^{\mathbf{o}}_{\downarrow,k}$ is the k-th column of $\mathbf{T}^{\mathbf{o}}$, so that $\boldsymbol{\mathcal{F}}\boldsymbol{\mathcal{D}}(\boldsymbol{\zeta})\mathbf{T}^{\mathbf{o}}_{\downarrow,k}$ is the wavefront arriving to the sensor from o_k. The symbol $\propto$ in Eq. (3) denotes equality up to a scale factor equivalent to the fluorescent efficiency. Also

in Eq. (3) α denotes the type of fluorescent excitation. The simplest case $\alpha = 1$ is known as single-photon fluorescence where the emission is linear in the excitation energy $|\boldsymbol{\nu}_k|^2$. In two-photon fluorescence, $\alpha = 2$, namely the emission is proportional to the squared excitation.

Phase conjugation: When the incoming and outgoing light have the same wavelength, the Helmholtz reciprocity principle leads to wave conjugation. Namely, if we record the wavefront emitted from a source point inside the tissue and illuminate with the conjugate wavefront, light will focus at the same point. This implies that the returning transmission matrix is the transpose of the incoming one [1]:

$$\mathbf{T^o} = \mathbf{T^i}^\top. \tag{4}$$

In the fluorescent case, $\mathbf{T^i}$ and $\mathbf{T^o}$ describe propagation at two different excitation and emission wavelengths. However, for linear single-photon fluorescence the excitation and emission wavelengths are relatively similar and $\mathbf{T^o} \approx \mathbf{T^i}^\top$. While our algorithm *does not* require the incoming and outgoing transmission matrices to be the same, this similarity will help us draw intuition on what is being optimized.

Normalization: we assume for simplicity that our transmission matrices are normalized such that every column or row has a unit energy. That is, for every k

$$\sum_x |\mathbf{T^o}_{x,k}|^2 = 1, \quad \sum_x |\mathbf{T^i}_{k,x}|^2 = 1. \tag{5}$$

This means that the total amount of energy that can arrive to particle o_k or emerge from it is fixed. Since the laser energy is fixed, we also assume w.l.o.g. that all illumination vectors have a unit norm $\|\mathbf{u}\| = 1$. As propagation through the tissue does not generate new energy, every incoming vector $\mathbf{u}$ should satisfy $\|\mathbf{T^i u}\| \leq 1$ and thus the energy at the target is also bounded:

$$\sum_k |\boldsymbol{\nu}_k|^2 \leq 1. \tag{6}$$

1.2 Score functions

We provide a longer derivation of the modulation scores mentioned in the main paper and explain how they can evaluate focusing.

The total intensity score: Consider a configuration where we only try to correct the illumination arm, and the SLM in the imaging arm is not used (equivalently, $\boldsymbol{\mathcal{D}}(\boldsymbol{\zeta})$ in Eq. (3) is the identity matrix). One of the earlier scores that were considered in the literature [2, 3] is just the total intensity measured over the entire sensor plane. Using Eqs. (3) and (5) it is easy to show that this total intensity score reduces to

$$\mathcal{S}_{\text{TI}}(\mathbf{u}) \equiv \sum_x \mathbf{I}(x) \propto \sum_k |\boldsymbol{\nu}_k|^{2\alpha}. \tag{7}$$

Since the energy at the target is bounded (see Eq. (6)), for the case $\alpha > 1$ this score is maximized when $\boldsymbol{\nu}$ is a one-hot vector, which equals 1 at a single entry and zero at all the others. However, in the single-photon case where $\alpha = 1$, Eq. (7) reduces to the total power in $\boldsymbol{\nu}$, $\mathcal{S}_{\text{TI}}(\mathbf{u}) = \sum_k |\boldsymbol{\nu}_k|^2$, and since this power is fixed, the same amount of energy returns whether we spread the excitation power over multiple fluorescence sources or bring all of it into one spot.

The variance maximization score: Boniface *et al.* [4] have recently suggested that to evaluate focusing with linear single-photon feedback, one should maximize the variance of the intensity measured by the sensor. The idea is that if we manage to focus all the excitation light at a single spot, the emitted light scattered through the tissue will generate a highly varying speckle pattern on the sensor plane. If the excitation is not focused, multiple sources emit simultaneously. The light emitted by these sources is summed incoherently, and hence the variance of the speckle pattern on the sensor decays. A short calculation provided in [4] shows

$$\begin{aligned}\mathcal{S}_{\text{Var}}(\mathbf{u}) \;&\equiv\; \text{Var}[\mathbf{I}] \equiv \frac{1}{n}\sum_x |\mathbf{I}(x)|^2 - \left(\frac{1}{n}\sum_x \mathbf{I}(x)\right)^2 \\ &\propto\; \sum_k |\boldsymbol{\nu}_k|^4,\end{aligned} \tag{8}$$

where n is the number of image pixels. Hence, as before, the score is a non-linear function of the power at different fluorescent particles and is maximized by a one-hot vector.

Confocal score: In our confocal system we use two modulations $\mathbf{u^i}, \mathbf{u^o}$ on the illumination and imaging arms and seek modulations that will maximize the energy at the central pixel. Below we derive the relation between the energy at the central pixel and the vector of fluorescent power $\boldsymbol{\nu}$, with the goal of showing that the confocal score is a non-linear function of the power of the excitation vector.

Denoting by $\boldsymbol{\mathcal{F}}_{0,\rightarrow}$ the central row of the Fourier transformation from the SLM plane to the image plane, the contribution of the particle o_k to our measurement at the central pixel is

$$\boldsymbol{\mathcal{F}}_{0,\rightarrow}\boldsymbol{\mathcal{D}}(\mathbf{u^o})\mathbf{T^o}_{\downarrow,k}. \tag{9}$$

Assuming w.l.o.g., that the central pixel is measuring the DC component of the Fourier transformation, corresponding to simple averaging, we can express the value at the central pixel in Eq. (9) as the product of the SLM modulation $\mathbf{u^o}$ at the imaging arm, with the corresponding column of the outgoing transmission matrix:

$$\boldsymbol{\mathcal{F}}_{0,\rightarrow}\boldsymbol{\mathcal{D}}(\mathbf{u^o})\mathbf{T^o}_{\downarrow,k} = \mathbf{u^o}^\top\mathbf{T^o}_{\downarrow,k}. \tag{10}$$

By modulating both illumination and imaging arms,

we can express the energy of the central pixel as:

$$\begin{aligned}\mathcal{S}_{\text{Conf}}(\mathbf{u^i}, \mathbf{u^o}) &\equiv \mathbf{I}(0) \propto \sum_k |\mathbf{u^o}^\top \mathbf{T^o}_{\downarrow,k}|^2 |\mathbf{T^i}_{k,\rightarrow}\mathbf{u^i}|^2 \\ &= \sum_k |\boldsymbol{\nu}^{\mathbf{o}}_k|^2 \cdot |\boldsymbol{\nu}^{\mathbf{i}}_k|^2. \end{aligned} \quad (11)$$

with $\boldsymbol{\nu}^{\mathbf{i}} = \mathbf{T^i}\mathbf{u^i}$ and $\boldsymbol{\nu}^{\mathbf{o}} = \mathbf{u^o}^T\mathbf{T^o}$. As mentioned in Eq. (6), the energy of $\boldsymbol{\nu}^{\mathbf{i}}$ is bounded, and due to reciprocity the same applies for $\boldsymbol{\nu}^{\mathbf{o}}$. It is easy to see that this score is maximized when $\boldsymbol{\nu}^{\mathbf{i}}, \boldsymbol{\nu}^{\mathbf{o}}$ are both one-hot vectors at the same entry k. That is, the score of Eq. (11) is maximized when the excitation modulation $\mathbf{u^i}$ brings all light to one of the particles o_k, and the modulation at the imaging arm $\mathbf{u^o}$ corrects the wavefront emitted from the same particle o_k and brings all of it into the central pixel.

In case $\mathbf{T^o} = \mathbf{T^i}^\top$ (so emission and excitation wavelengths are similar) it can be shown that the optimal solution is obtained by $\mathbf{u^o} = \mathbf{u^i}$, namely, when using the same modulation at both the illumination and imaging arms, and the score of Eq. (11) reduces to $\sum_k |\boldsymbol{\nu}_k|^4$ as in the variance-maximization and two-photon cases.

Optimization: In this work we have explicitly optimized the confocal score (Eq. (11)) using standard Hadamard basis optimization [5], detailed in Sec. 2. Overall the Hadamard optimization is significantly slower than [6]. We note that when all fluorescent sources o_k have the same power, there are K different solutions that can maximize Eq. (11) and an optimization may converge to any of them. Also note that we constrain the solution such that regardless of the position of o_k, it will be imaged in the central pixel. If o_k is not at the center of the frame, the wavefront $\mathbf{u^o}$, placed at the Fourier plane of the imaging system will contain a tilt, shifting o_k to the central pixel.

2 Optimization algorithm

This section provides details on our optimization algorithm. In Sec. 4 below we compare two different correction models. (i) Assumes that as our excitation and emission wavefronts are sufficiently similar, and use the same modulation $\mathbf{u}$ in both the illumination and imaging arms. (ii) Solve for a different modulation in each arm. We start with the first case.

2.1 Same modulation in both arms

Assuming the excitation an emission wavelengths are sufficiently similar $\mathbf{T^o} \approx \mathbf{T^i}^\top$ the confocal score derived in Eq. (11) reduces to:

$$\begin{aligned}\mathcal{S}_{\text{Conf}}(\mathbf{u}) \propto \sum_k |\mathbf{u}^T\mathbf{T}^{\mathbf{o}}_k|^2 \cdot |\mathbf{T}^{\mathbf{i}}_k\mathbf{u}|^2 = \\ = \sum_k |\mathbf{T}^{\mathbf{i}}_k\mathbf{u}|^4. \end{aligned} \quad (12)$$

where $\mathbf{T}^{\mathbf{i}}_k$ is the k'th row of the input transmission matrix from the illumination SLM to the particles, $\mathbf{T}^{\mathbf{o}}_k$ is the k'th column of the output transmission matrix from the particles to the imaging SLM and $\mathbf{u}$ is the wavefront correction vector on both SLMs.

Our optimization scans a set of binary phase masks and for each of them finds the phase that maximizes the score function. We show below that if only the phase of this mask can be adjusted, the score can be expressed as the sum of two sinusoidal functions, which can be measured using 5 samples. By capturing 5 shots we can select the optimal value for this phase and proceed to the subsequent mask.

Dictionary representation: In our optimization, we mark the phase function on the SLM in the mth iteration as $\boldsymbol{\psi}_{(m)}(\omega)$. We express the phase mask as a superposition of a dictionary of N binary masks, weighted by phases ϕ_n:

$$\boldsymbol{\psi}_m(\omega) = \sum_{n=1}^{N} \phi_n \cdot \boldsymbol{\mu}_n(\omega). \quad (13)$$

Here ϕ_n is a scalar coefficient, $\boldsymbol{\mu}_n(\omega)$ is a binary vector whose size is equivalent to the number of entries in the SLM, and ω is the index of an entry in the SLM plane. Since we use phase-only SLMs, the amplitude in each pixel remains constant and the wavefront correction vector is expressed as:

$$\mathbf{u} = \exp\{i\boldsymbol{\psi}_m(\omega)\} = \exp\left\{ i \sum_{n=1}^{N} \phi_n \cdot \boldsymbol{\mu}_n(\omega) \right\}. \quad (14)$$

In the mth iteration of our optimization, we choose a single dictionary element n and update ϕ_n to obtain:

$$\boldsymbol{\psi}_m(\omega) = \boldsymbol{\psi}_{m-1}(\omega) + \phi_n \cdot \boldsymbol{\mu}_n(\omega), \quad (15)$$

Sinusoidal model:

Claim 1. *If we only vary the scalar ϕ_n the confocal score of Eq. (12) can be expressed as a sum of two sinusoids*

$$\mathcal{S}_{Conf}(\phi_n) = |A| + |B|\cos(\phi_n + \angle B) + |C|\cos(2\phi_n + \angle C), \quad (16)$$

where A, B, C are complex scalars, and we denote their amplitude and phase by $|A|, |B|, |C|$, $\angle A, \angle B, \angle C$ respectively.

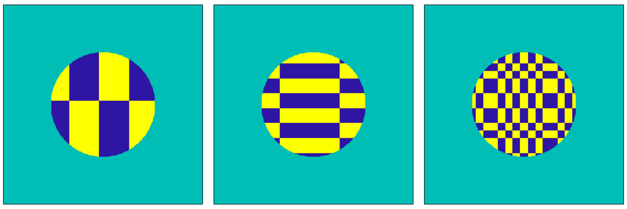

Fig. 1: Visualizing a few Hadamard masks. The masks are placed in the Fourier plane and they are all cropped to the circular aperture area. In the figure, the leftmost mask has low frequencies, while the rightmost one corresponds to higher frequencies.

Proof. We consider a binary mask $\boldsymbol{\mu}_n(\omega)$, and denote its complimentary by $\overline{\boldsymbol{\mu}_n(\omega)}$. With this notation we can express the modulation $\mathbf{u}$ of Eq. (14) as

$$\begin{aligned} \mathbf{u}(\phi) &= e^{i\boldsymbol{\psi}_m(\omega)} = e^{i(\boldsymbol{\psi}_{m-1}(\omega)+\phi\cdot\boldsymbol{\mu}_n(\omega))} \\ &= e^{i\boldsymbol{\psi}_{m-1}(\omega)}\overline{\boldsymbol{\mu}_n(\omega)} + e^{i\phi}e^{i\boldsymbol{\psi}_{m-1}(\omega)}\boldsymbol{\mu}_n(\omega). \end{aligned} \tag{17}$$

We mark

$$\begin{aligned} \mathbf{u}^{(1)} &= e^{i\boldsymbol{\psi}_{m-1}(\omega)} \cdot \overline{\boldsymbol{\mu}_n(\omega)}, \\ \mathbf{u}^{(2)} &= e^{i\boldsymbol{\psi}_{m-1}(\omega)} \cdot \boldsymbol{\mu}_n(\omega), \end{aligned} \tag{18}$$

and so, $\mathbf{u}(\phi)$ simplifies to:

$$\mathbf{u}(\phi) = \mathbf{u}^{(1)} + \mathbf{u}^{(2)} \cdot e^{i\phi}. \tag{19}$$

With this notation we can express

$$\boldsymbol{\nu}_k = \mathbf{T}_k^{\mathbf{i}}\mathbf{u}(\phi) = v_k^{(1)} + v_k^{(2)}e^{i\phi}, \tag{20}$$

with $v_k^{(1)} = \mathbf{T}_k^{\mathbf{i}}\mathbf{u}^{(1)}$ and $v_k^{(2)} = \mathbf{T}_k^{\mathbf{i}}\mathbf{u}^{(2)}$. A short calculation shows that $|\boldsymbol{\nu}_k|^4$ follows the form of Eq. (16). The confocal score of Eq. (12) is equivalent to $\sum_k |\boldsymbol{\nu}_k|^4$, and hence, when considering a binary basis element the change to the score can be expressed as a sum of two sinusoids. □

Optimization details: Using the above claim, we can consider $J \geq 5$ equally spaced phases $\phi^j = [1\ldots J]\frac{2\pi}{J}$, generate the wavefronts $\boldsymbol{\psi}_m(\omega)^j = \boldsymbol{\psi}_{m-1}(\omega) + \phi^j \cdot \boldsymbol{\mu}_n(\omega)$ and place them on both SLMs. For each phase we measure the intensity at the central pixel. We use these J intensity measurements to fit a sinusoid and find the value ϕ_n maximizing Eq. (16).

In practice we notice that $|C|$ is usually smaller than $|A|, |B|$ and it is enough to fit the score function as a single sinusoid, hence we can also reduce the number of samples to $J = 3$ or $J = 4$.

Binary masks: We divide the SLM to $\sqrt{N} \times \sqrt{N}$ super-pixels, and choose to use binary Hadamard masks as our dictionary. We show several such Hadamard masks in Fig. 1. The advantage of this dictionary is that the ϕ_n we adjust in each iteration is displayed on half of the SLM area, rather than on a single pixel. Thus, it has more impact on the intensity we measure and the measurement is less sensitive to noise.

Since the speckles from a single source have a compact support, as demonstrated e.g. in Fig. 3 of the main paper, we conclude that the correction mask has more content at the lower frequencies and less content on the higher ones. To account for that, we first scan the Hadamard basis elements that correspond to low frequencies and later the Hadamard elements with higher frequency content. We return to the same dictionary element more than once during optimization, and we invest more optimization iterations in the low frequency elements.

2.2 Different modulations in illumination and imaging arms

So far we considered a model constraining the modulations on both SLMs to be the same. This is only an approximation to the desired modulation, because the illumination and excitation wavelengths can differ. To solve for two different modulations we alternate between updating the ϕ_n values in the excitation modulation, and updating the ϕ_n values of the emission modulation. If we fix one modulation and vary the other, the confocal score $\mathcal{S}_{\text{Conf}}(\phi_n)$ is a single sinusoid rather than a sum of two sinusoids as in Eq. (16), and its phase can be fitted with $J = 3$ samples. In Sec. 4 below we compare two different modulations to a single modulation in both arms. We find out that two different modulations can lead into a somewhat better correction, but this optimization also doubles the number of exposures. Therefore, in the presence of photo-bleaching, a single modulation usually leads to better results.

3 Additional results

In Fig. 3 we show additional images of the focused spot achieved with our modulation. In particular, in the last row we show one failure example where the algorithm has converged on two spots rather than one.

Additional results imaging a thin brain layer with neurons behind chicken breast or parafilm layers are presented in Figs. 4 and 5. While the modulation we recover focuses at a single spot, due to the memory effect we can use it to image an area behind the tissue rather than a single spot. In Sec. 6 below we explain the tilt-shift adjustments required.

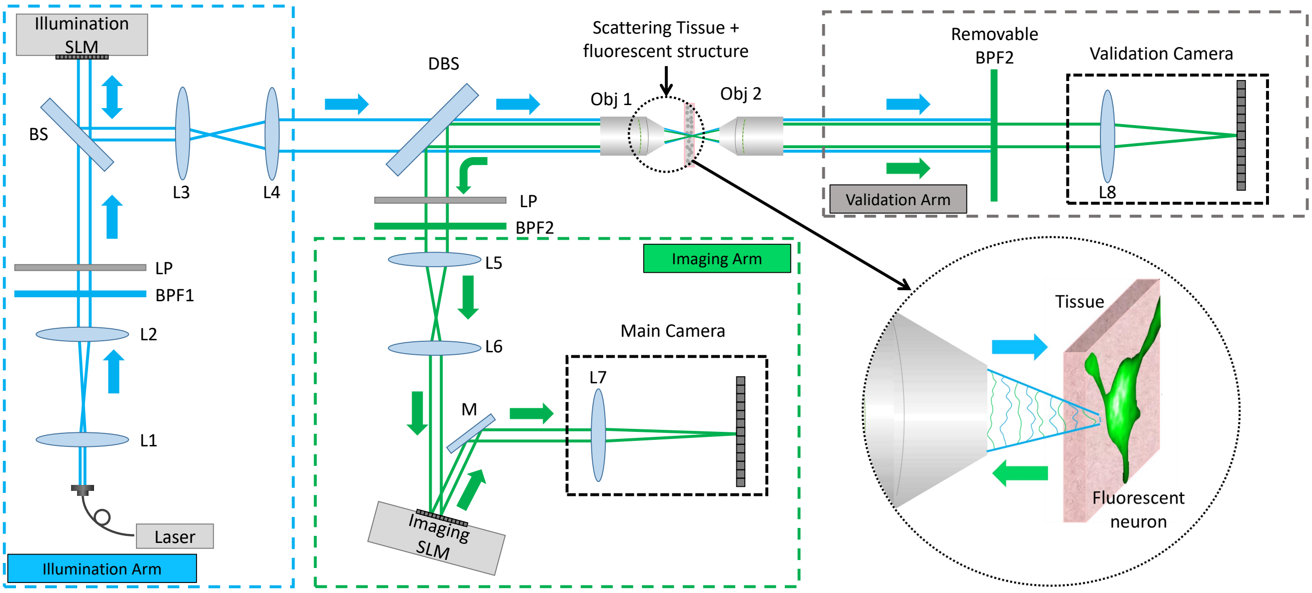

Fig. 2: Imaging setup: A laser beam is exciting a fluorescent target at the back of a tissue layer, and fluorescent emission is scattered again through the tissue, reflects at a dichroic beam-splitter and is collected by a main (front) camera. We place two SLMs in the Fourier planes of both illumination and imaging arms to allow reshaping these wavefronts. A validation camera views the fluorescent target at the back of the tissue directly. This camera is not actually used by the algorithm, and is only assessing its success. LP=linear polarizer, BS=beam-splitter, DBS=dichroic beam-splitter, BPF=bandpass filter, M=mirror, $L1 \dots L8$=lenses, Obj=Objective. Components are listed in Table 1.

Laser	Coherent Sapphire 488-200CW
LP	Linear polarizer, Thorlabs LPVISE200-A
BS	Beam-splitter, Edmund Optics 68413
M	Mirror, Thorlabs PFR10-P01
DBS	Dichroic beam-splitter, Thorlabs DMSP490
BPF1	Illumination bandpass filter, Edmund Optics 65147
BPF2	Imaging bandpass filters, Edmund Optics 67030 + 65151
L1	Plano convex, 4cm
L2	Plano convex, 40cm
L3	Plano convex, 10cm
L4,L5,L8	Tube lense 20cm, Thorlabs TTL200
L6	Plano convex, 12.5cm
L7	Plano convex, 15cm
Objectives	Thorlabs N20X-PF
Main camera	Teledyne Photometrics Prime BSI Express
Validation camera	Flea3 FL3-G3-28S4M
SLM	Holoeye Pluto

Table 1: **List of components.**

In Fig. 6 we show additional results imaging through a thick $400\mu m$ brain slice. Since the fluorescent target has 3D variation, we use a confocal scanning to isolate a neuron at a single depth. A confocal scanning of our modulation is significantly better than an uncorrected confocal scan. Also due to the 3D structure it is not always possible to capture a good full-frame reference from the validation camera.

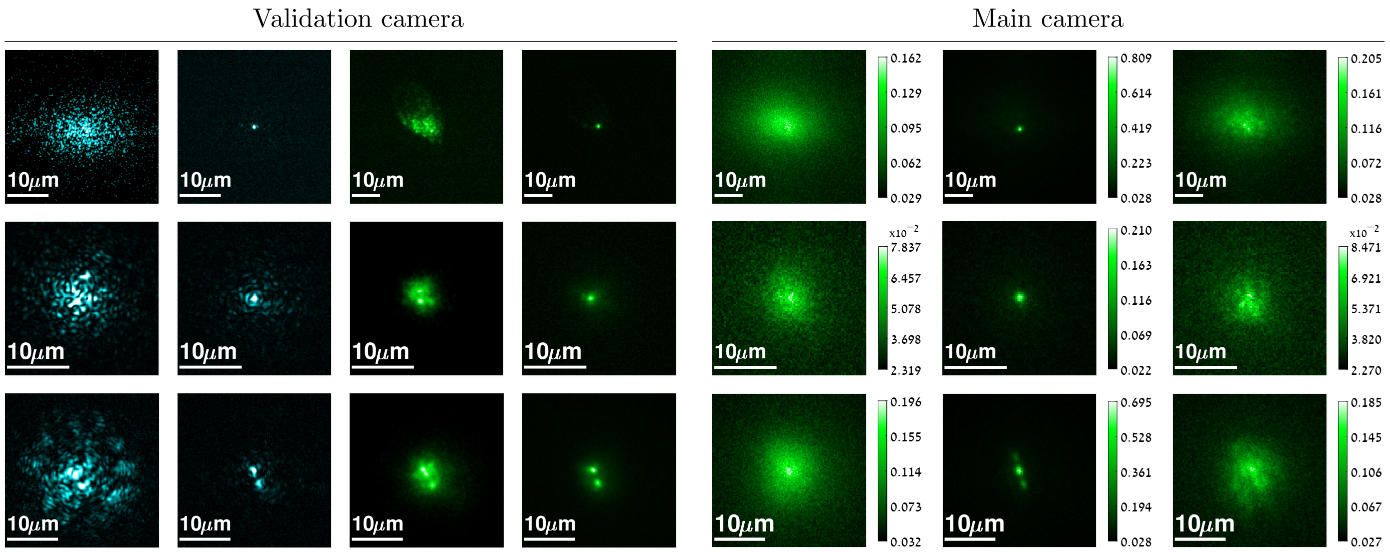

(a) No mod. (b) With mod. (c) No mod. (d) With mod. (e) No mod. (f) With mod. (g) PSF

Excitation Emission Emission

Fig. 3: Additional wavefront shaping results: we visualize views from the validation and main cameras. (a-b) The excitation light as viewed by the validation camera at the back of the tissue. Due to significant scattering, at the beginning of the algorithm when no modulation (mod.) is available, a wide speckle pattern is generated. After optimization, the modulated wavefront is brought into a single spot. (c-d) By placing a band-pass filter on the validation camera, we visualize the emitted light with and without the modulation correction. (e-f) Views of the emitted light at the main front camera with and without the modulation correction. Note that this is the only input used by our algorithm. Without modulation, light is scattered over a wide image area and the image is noisy. A sharp clean spot can be imaged when the limited number of photons is brought into a single sensor pixel. (g) By correcting the emission such that a single spot is excited and leaving the imaging path uncorrected, we can visualize the actual aberration of a single fluorescent point source. Each row demonstrates a different tissue sample. The top example demonstrates a thin brain layer behind parafilm, and the two lower ones are from a thick brain slice. The lowest row demonstrates a failure example where the optimization converged at two spots rather than one.

In Fig. 7 we test the actual extent of the memory effect for such samples. We run the algorithm until convergence and then start to tilt-shift the modulation so that the focal spot translates along a line at the back of the tissue. We capture the intensity of the translating spot from the validation camera and plot it as a function of distance from the original focal spot. To demonstrate the variance of such curves we plot 4 curves shifting the point at 4 different directions. We include two examples, one focusing through a $400\mu m$ brain slice and one focusing through a layer of parafilm. We include a few insets visualizing how speckles around the focal spot increase as it is translates.

4 One modulation against two modulations

As mentioned above, in our single-photon fluorescence case the emission and excitation wavelengths are similar and we can approximately use the same modulation in both excitation and emission arms. Alternatively we can solve for a different modulation in each arm, but this doubles the number of exposures. Below we compare these two approaches experimentally and learn that while two different modulations can lead to a better correction, they also require a longer acquisition with more bleaching. In the presence of bleaching the faster, one-modulation approach usually leads to better results.

Comparing two algorithms on the same sample is challenging, because if we try to run two algorithms sequentially on the same sample, the second one would have worse results just because more bleaching took place. To avoid this we run the two algorithms in alternating order. The m'th iteration is composed of 4 steps:

1. Test basis element $2m$ for the single modulation case.
2. Test basis element m for the excitation arm in the two-modulation case.
3. Test basis element $2m+1$ for the single modulation case.

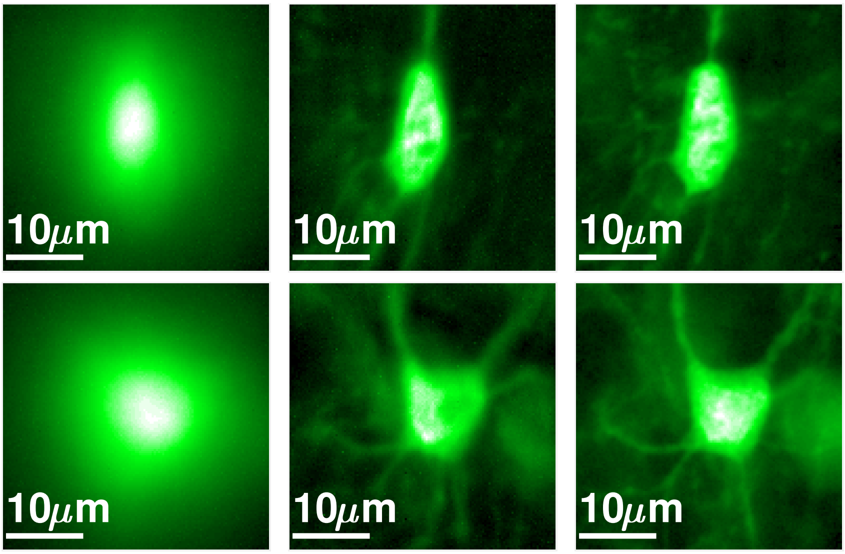

(a) Uncorrected Main camera | (b) Corrected Main camera | (c) Reference Validation camera

Fig. 4: Additional results: a thin brain layer behind chicken breast tissue. (a) Image of the neuron from the main camera with no correction, strong scattering is present and the neuron structure is lost. (b) Image with our modulation correction, the neuron shape as well as some of the axons are revealed. (c) A clean reference image of the same neuron, from the validation camera.

4. Test basis element m for the emission arm in the two-modulation case.

The implication is that the single modulation approach scans the elements of the Hadamard basis faster than the two-modulation approach. In the results of Fig. 8 we scan the Hadamard basis 4 times in the single modulation optimization and only twice in the two-modulation optimization. We separate the measurement of the single and two-modulation approaches and plot them as two separate curves in Fig. 8(a). The single modulation approach leads to higher energy at the focused spot. To evaluate the quality of the modulation independently of bleaching, at the end of the optimization we capture 3 images of the focus spot. 1) With two different modulations, scanning each basis element twice. 2) With a single modulation with the same number of exposures, effectively scanning each element 4 times. 3) An image of a single modulation in the middle of the optimization, which effectively scans the basis elements only twice as the two-modulation optimization. While this image corresponds to the modulation in the middle of the optimization, we re-capture it at the end, in an equivalent bleaching stage. The single modulation that scanned the basis elements 4 times gives the best results. However if we compare the two modulations to a single modulation that has scanned the elements for the same number of times, the results are better.

In Fig. 8 we demonstrate this comparison twice, once when the fluorescent target is a neuron behind a layer of parafilm, and once when we spread a set of fluorescent beads behind the parafilm. In Fig. 8(f-g) we show images of the target from the validation camera in the beginning of the optimization and at the end. For the beads example, one can see that the bead at which the algorithm has converged (marked by an arrow) is significantly dimmer at the end of the optimization, showing the strong bleaching.

In Fig. 9 we show the phase of the modulation masks we found with the different approaches described above. The correlation between the modulations at the two different wavelengths is typically within the range $[0.6, 0.8]$. The correlation between modulations found at two different wavelengths to the same modulation for both wavelengths is also in the same range. It is unclear if this is really a measure of the chromatic memory effect correlation, or if this difference is a result of the noisy optimization.

5 Comparison with alternative wavefront-shaping scores

We compare our confocal score with the variance maximization approach of [4], showing that our approach can converge using a significantly smaller number of photons.

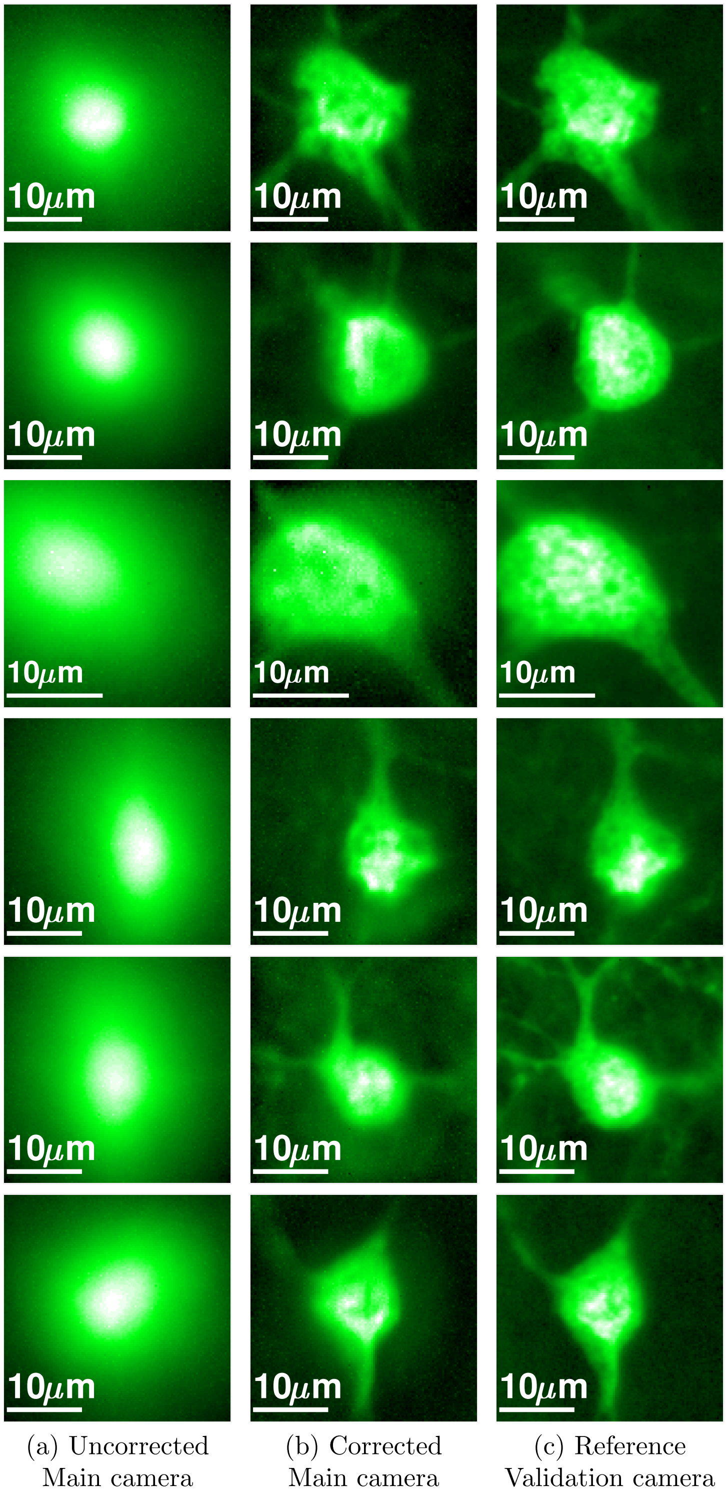

Fig. 5: Additional results: a thin brain layer behind parafilm. (a) Image of the neuron from the main camera with no correction, strong scattering is present and the neuron structure is lost. (b) Image with our modulation correction, the neuron shape as well as some of the axons are revealed. (c) An undistorted image of the same neuron, from the validation camera.

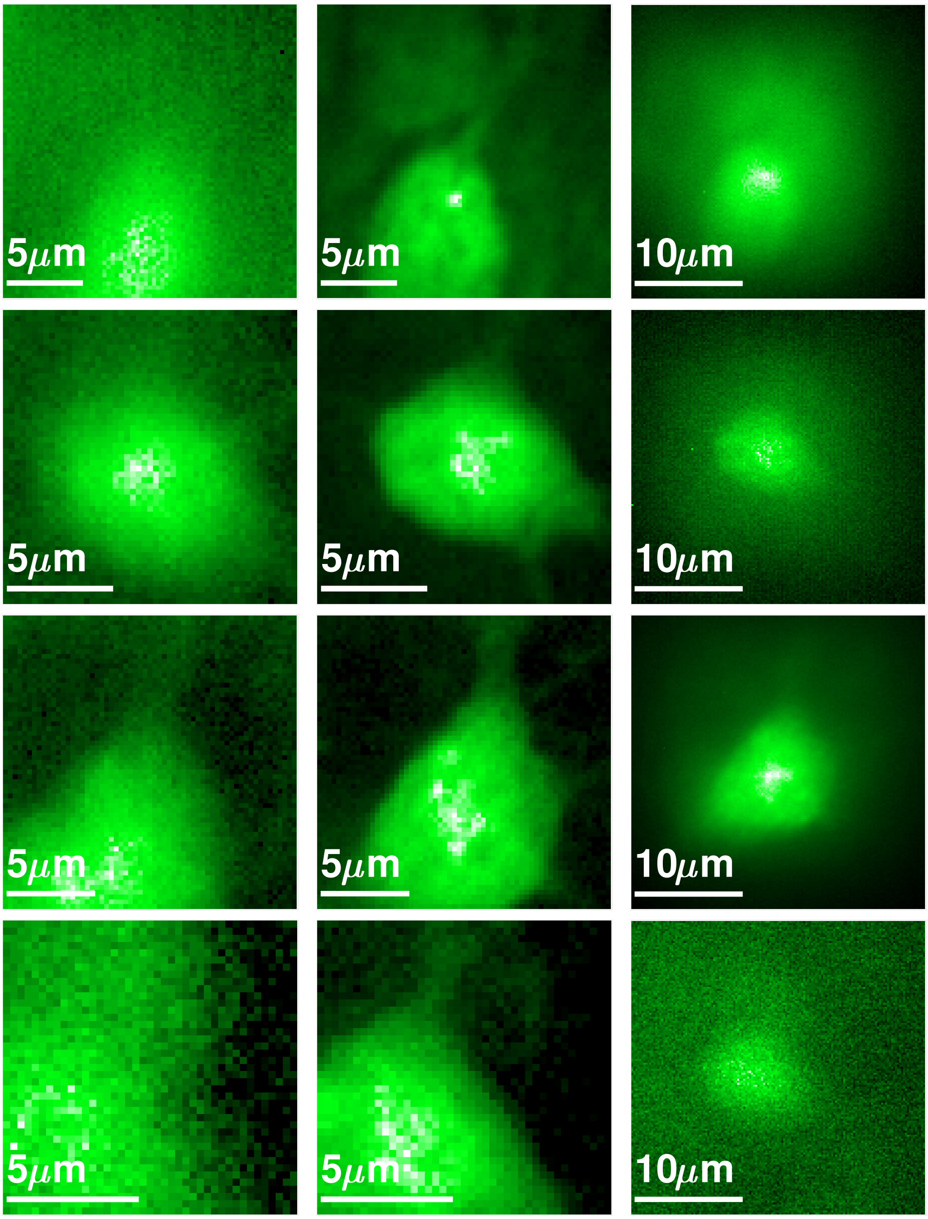

(a) Uncorrected confocal
Main camera

(b) Corrected confocal
Main camera

(c) Reference
Validation camera

Fig. 6: Additional results: imaging inside a 400μm thick fluorescent brain slice. (a) A confocal image of the neuron from the main camera with no correction, strong scattering is present and the neuron structure is lost. (b) A confocal image with our modulation correction, the neuron shape as well as some of the axons are revealed. (c) A reference image of the same neuron, from the validation camera. Due to the 3D spreading of the fluorescent components, the validation camera cannot always capture an aberration-free image of the target.

We also compare against one of the non-local approaches in [7]. This approach assumes that a single modulation can correct a wide image region rather than a single spot. Our evaluation shows that when memory-effect exists over a wide extent this algorithm can indeed recover good modulations, but the quality of the results degrades for a short ME, where the size of the iso-planatic patches that can be corrected with a single modulation is small.

We note that it is very hard to run two algorithms on the same data under equal noise conditions, as due to bleaching the second algorithm would run on a significantly weaker fluorescent source. To support a controlled

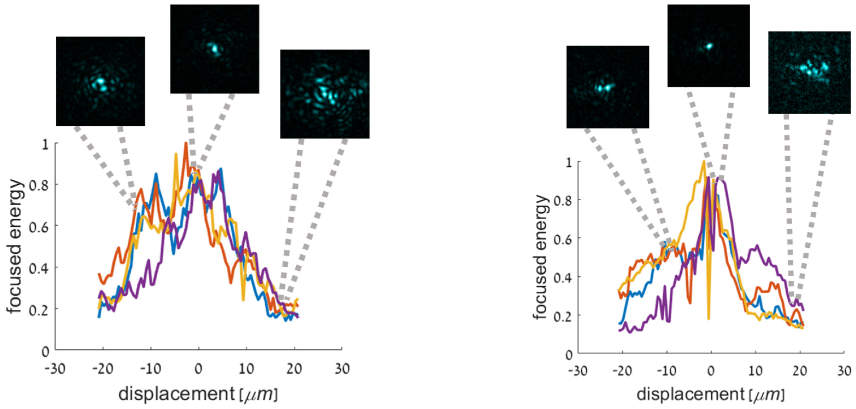

Fig. 7: Extent of memory effect correlation: we plot the decay of memory effect correlation in our samples, by translating a recovered modulation to nearby points and measuring the energy of the focused spot behind the tissue, at the validation camera. Insets show sample shapes of the focused spots. As translation is larger, more speckles arise around the desired focused spot. We plot 4 different line scans in different orientations to demonstrate the variance of such curves. Left: focusing through a 400μm brain slice. Right: focusing through a layer of parafilm.

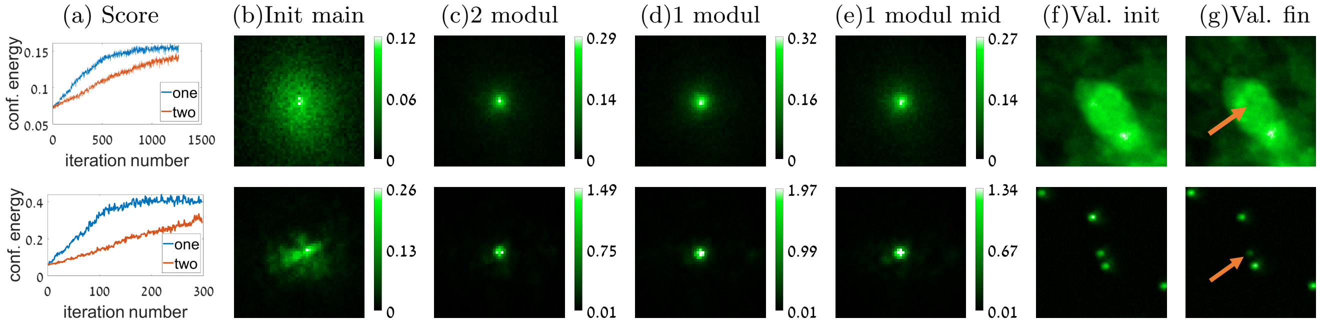

Fig. 8: Testing the single modulation approximation: We compare the usage of two different modulations on the emission and excitation wavelengths, vs. a common modulation for both. The top row shows results on a brain slice and the lower one uses as the target a set of fluorescent beads behind parafilm. (a) The confocal intensity at the central pixel as a function of the number of iterations. Since the two-mask approach doubles the number of measurements for each dictionary element, its convergence is slower. (b) The initial image at the main camera. (c) The final confocal spot at the main camera using 2 different modulations. (d) The final spot using the same modulation in both channels. Since it scans the dictionary elements more times, the result is better (a higher energy is measured at the central pixel). (e) The result of one mask in the middle of the optimization when the number of dictionary elements is equivalent to what the two-modulation approach has scanned at the end of the optimization. When scanning the dictionary for the same number of times, the one-modulation result is somewhat lower than what was achieved by two modulations. (f-g) The fluorescent target from the validation camera at the beginning of the optimization and at the end, notice the strong bleaching at the focusing bead, marked with an arrow. We show the final modulations in Fig. 9.

evaluation we used simulated transmission matrices synthesized using a multi-plane propagation model. We assume the tissue has a thickness of $L = 250\mu m$ and the aberration of light propagating through this volume is formed by equally spaced, planar pseudo-random phase masks. We use different number of aberration layers to test different memory-effect extents as described below. Given a transmission matrix we can simulate the images formed under any modulation of choice and add a different amount of noise. Hence we can evaluate the results of different algorithms while varying any parameter of interest.

5.1 Speckle-variance maximization

As mentioned in the main paper both our confocal score and the variance maximization score seek to optimize the same non-linear function of fluorescent intensity, yet they are not equivalent in terms of SNR. To demonstrate

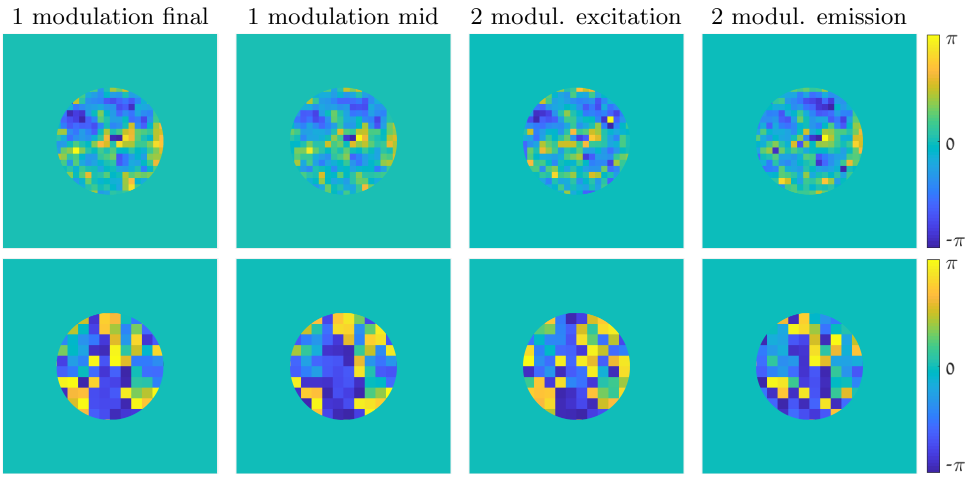

Fig. 9: Modulation masks: We visualize the modulations resulting from Fig. 8, in the Fourier plane. The correlation between the masks of the two different wavelengths is typically within the range $[0.6, 0.8]$. The correlation between the one-mask approach and the two-mask approach is also in the same range.

this we run both algorithms, and in each iteration, as we update the modulation we also vary the laser power such that the SNR of the score we measure in each algorithm will be kept fixed. Since our algorithm attempts to bring all photons to one sensor pixel, with the same laser power, the images we measure are less noisy then speckle-variance score (which does not use a correction on the emitted speckles). Equivalently, we can achieve the same SNR as the speckle variance score with a weaker excitation power.

Fig. 10(d) demonstrates the curve of laser power as a function of iteration number in each algorithm. One can see that our algorithm can work with a significantly lower number of photons, meaning that the algorithm has a much better chance to converge without much photobleaching. We have repeated the evaluation with three different transmission matrices, where we changed the statistics of the aberration layers to be less forward scattering, hence the speckle support is wider. We can see that for a wider aberration the gain of the confocal score is higher (compare the different rows of Fig. 10, for the small support we require 5.8× fewer photons, for the wide one we requiere 43× fewer photons). This is because for a wider speckle pattern the speckles are spread over more pixels, hence the images are noisier and estimating the speckle variance without modulating the emission path is harder.

5.2 Non-local scores

Next, we compare against a recent non-local approach[7]. This assumes that a single modulation can correct a wide image region rather than a single spot. It uses a wide illumination and only correct the imaging arm, where a good modulation should lead to a sparse image, measured using a combination of a modified entropy and a variance maximization scores.

In Fig. 11 we evaluate this approach using two different transmission matrices. In the first case we used a transmission matrix generated by a single aberration layer. In this case the memory effect correlation holds over a wide extent, and a single modulation can correct a wide isoplanatic region. In the second case we simulated a transmission matrix with 3 different aberration layers equally spaced between depth 0 to $250\mu m$. This is a more realistic approximation of a scattering tissue exhibiting volumetric aberration, but the extent of the memory effect is much shorter. In this case there is no single modulation which can fully correct the entire image. Fig. 11 shows a comparison of this algorithm against our confocal score with two structures of hidden neurons. When memory-effect exists over a wide extent the non-local score of [7] can indeed recover good modulations, but the quality of the results degrades when ME range is short, and the size of the iso-planatic patches that can be corrected with a single modulation is small.

6 Tilt-shift correction

Below we explain the acquisition of wide area images of Figs. 4 and 5. We follow the strategy used in [6]. Given a wavefront shaping modulation that applies to one fluorescent particle inside the tissue sample, we correct nearby ones using the tilt-shift memory effect. For that, we denote by $\mathbf{u}_x^{o_1}, \mathbf{u}_x^{o_2}$ two speckle fields obtained on the sensor

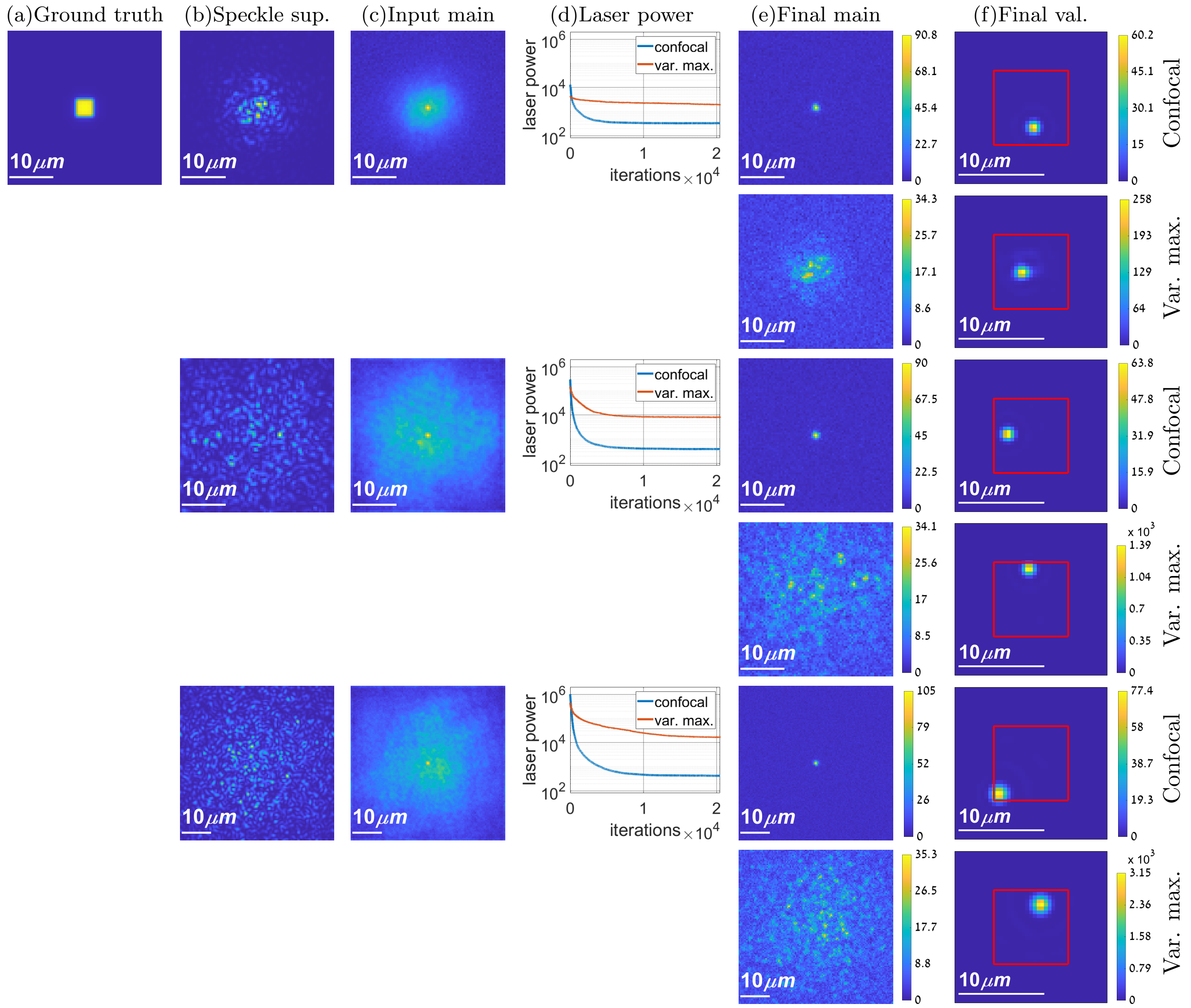

Fig. 10: Comparing the variance maximization score against the confocal score. During optimization we reduce the laser power so that the SNR of the captured images remains fixed. Using the confocal score, as the estimated modulation improves, more photons are brought into a single pixel and hence we can capture a high SNR image with a weaker laser power, reducing photo-bleaching. (a) Fluorescent target from the validation camera. (b) Scattering by a single spot (one row of the transmission matrix), illustrating the speckle spread. (c) Input image from the main camera. (d) Laser power at each iteration in both algorithms. (e) Final image from the main camera. The confocal algorithm results in a spot, and the variance maximization algorithm results in a speckle pattern. (f) Final image from the validation camera where both algorithms excite a single spot. The original area of the fluorescent target is marked with a square. The position of the focused spot inside the fluorescent area can vary. The different rows simulate 3 different speckle supports. When scattering is wider the advantage of the confocal score is more dominant, whereas for the smallest support we require 5.8× fewer photons and for the wider one 43×.

plane of our main camera (where x denotes spatial position on this plane), generated by fluorescent particles at o_1, o_2. We focus the objective such that the sensor plane is conjugate to the plane containing the fluorescent sources. The tilt-shift memory effect [8, 9] implies that for small displacements, u^{o_1} is correlated with a tilted and shifted version of u^{o_2} and thus can be approximated as:

$$\mathbf{u}_x^{o_1} \approx \mathbf{u}_{x+\mathbf{\Delta}}^{o_2} \cdot e^{iks<\mathbf{\Delta},x>} \tag{21}$$

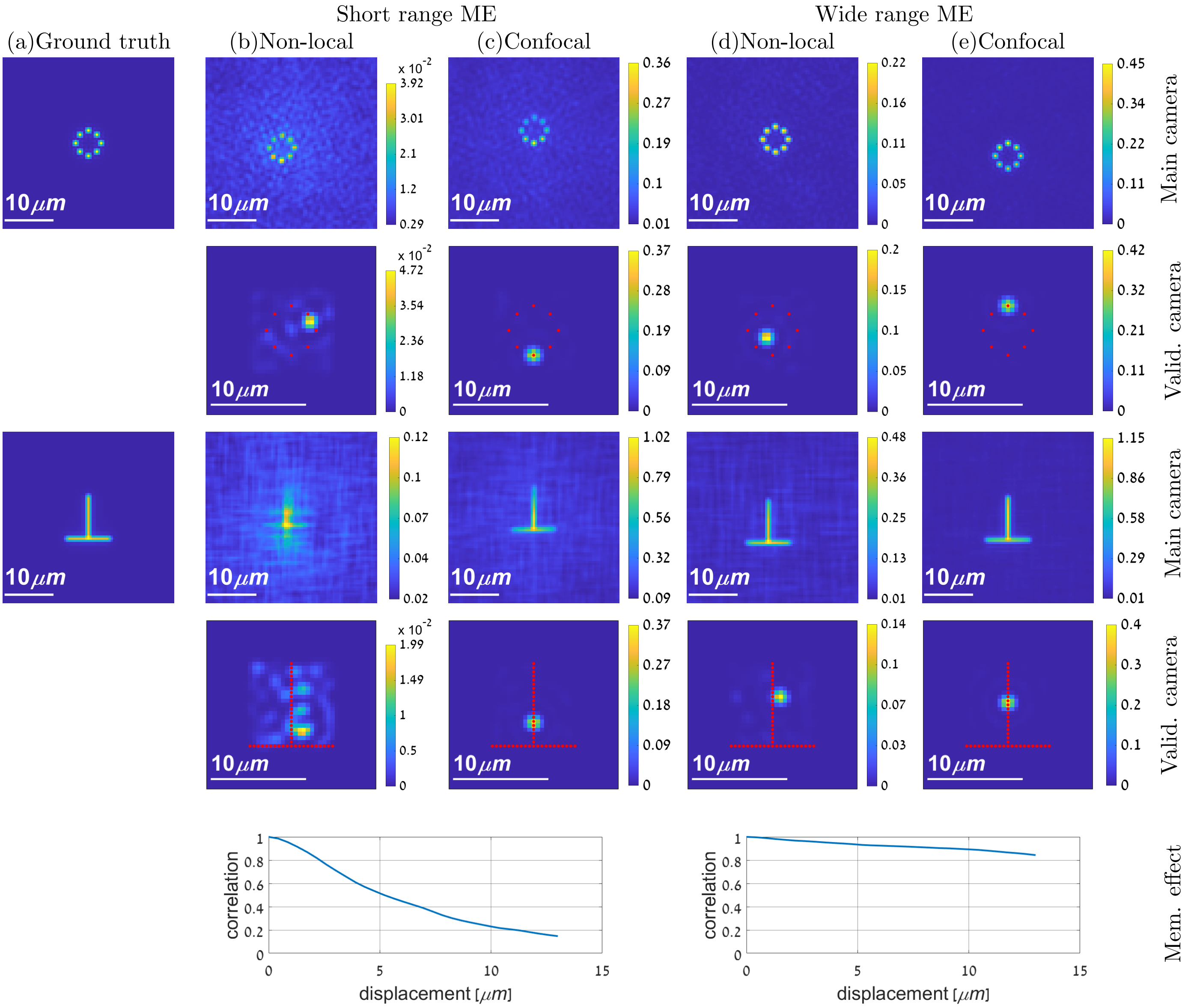

Fig. 11: Comparison against a non-local score [7]. (a) The ground truth fluorescent target. (b-c) Results using a transmission matrix with short range memory effect. In this case a single correction cannot explain the full image and the non-local approach leads to degraded results. (e-f) Results in the presence of long-range memory effect, where the non-local approach is successful. We simulate two fluorescent targets, the top one is sparser and easier to handle and the lower one is denser. In each example the top row shows images from the main camera under a wide illumination (for the confocal algorithm, we run it until convergence and then use a wide illumination while only correcting the imaging arm). In the lower row we use the recovered modulation in the imaging arm and show a view from the validation camera. If the modulation is good it should bring all light into a single sharp spot. The overlaid red dots illustrate the actual position of the fluorescent target. The lowest row shows a plot of the decay of memory effect correlation in each of the transmission matrices.

with $\mathbf{\Delta} = o_2 - o_1$ the displacement between the sources, and s is scaling the tilt according to the thickness of the sample. The scale s can be derived from various geometric considerations [8, 9], but in practice we fit its value as described in Sec. 7 below. If there was no tilt, and the speckle at the image plane could be explained by pure shift, placing in the Fourier plane the Fourier transform of $u_x^{o_1}$ would correct the emission from o_1 and the emission from nearby points o_2. Given the tilt, the Fourier correction for o_2 should be a shifted version of the Fourier cor-

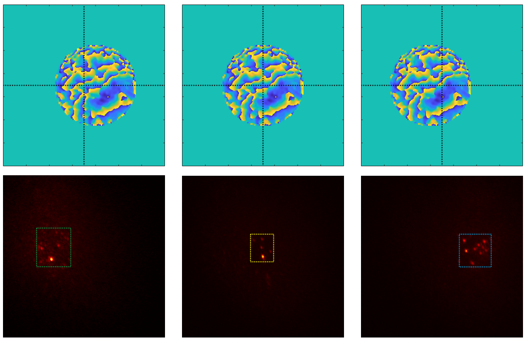

(a) Shifted correction patterns and resulting images.

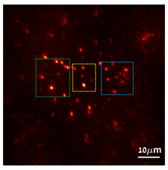

(b) Combined

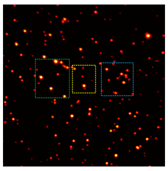

(c) Reference

Fig. 12: Using the tilt-shift memory effect to see a wide area behind the tissue. (a) Ee demonstrates three different shifts of the recovered correction pattern, and below them the image captured by placing this shifted mask on the SLM of the imaging arm. Each shift allows us to see a different sub-region of fluorescent sources. By merging 21 × 21 such shifts we get the wider image in (b). (c) We compare the reconstruction against a reference from the validation camera.

rection of o_1. To account for this, we place in the Fourier plane of our imaging arm shifted versions of our recovered mask. Fig. 12 illustrates that using a fluorescent beads target. Each shift allows us to see the fluorescent particles in a different local region. By scanning multiple shifts of the modulation mask, we construct a wider image of the fluorescent particles inside the tissue sample, as shown in the last row of Fig. 12.

7 Calibration and alignment

Below we elaborate on various calibration and alignment details.

First, to correctly modulate the Fourier transform of the wave, the illumination SLM needs to be at the focal plane of the lens right after it ($L3$ in the system figure), and the imaging SLM at the focal plane of the lens before it ($L6$). We do this alignment using another camera focused at infinity (we focus this camera by imaging a far building). We use this camera to view the SLM through the relevant lens, forming a relay system. We adjust the distance between the SLM and the lens until the calibration camera can see a sharp image of the SLM plane. We also ensure that the distance between the sensor of the main/validation cameras and the lenses $L7/L8$ attached to them is set such that the cameras focus at infinity.

A second step of the alignment is to focus the excitation laser and the system camera on the same target plane. In our setup the sample and the objective of the validation camera are mounted on two motorized z-axis (axial) translation stages. We use fluorescent beads with no aberrating tissue and adjust the axial distance between the beads and the objective of the main camera (Obj1 in the setup figure) such that the main camera sees a sharply focused image of the bead. Then we adjust the distance of the validation objective (Obj2 in the setup figure) from the beads so that we see a sharp image of the same beads in the validation camera. We then want the laser to generate its sharpest spot on the same plane. Assuming the validation and main camera are focused at the same plane, we adjust the position of the lens $L4$ until the validation camera sees a sharp laser spot.

After the system has been aligned, we need to determine two mappings. The first one is between frequencies to pixels on the SLM. A second, more challenging one is the registration between the two SLMs, so that we can map a pixel on the imaging SLM to a pixel on the illumination SLM controlling the same frequency. We start with a mapping between frequencies to the SLM on the imaging arm. We first put a calibration camera that can image the camera SLM plane directly when it receives laser light. Since the SLM is conjugate to the aperture of the objective we see an illuminated circle on the SLM plane, corresponding to the numerical aperture of the imaging system. The center of this circle gives us a first estimate of the zero (central) frequency of the Fourier transform. Using the focal length of $L6$, the SLM pitch and the wavelength of the emitted light, we can map frequencies to SLM pixels using simple geometry. Alternatively, we can display on the SLM sinusoidals of various frequencies. This shifts the image on the sensor plane. By measuring the shift resulting from each sinusoidal we can calibrate the mapping between frequencies and SLM pixels. To align between the two SLMs we place an isolated fluorescent bead behind a scattering tissue. We capture a few images of the speckles resulting from this bead (at emission wavelength) and use a phase diversity approach [10] to estimate the complex wavefront that emerges from this bead. The Helmholtz reciprocity (phase conjugation) principle states that if the conjugate of this wavefront is placed on the illumination SLM, it will focus into a point behind the tissue. However, we need to determine how to position this modulation on the SLMs keeping in mind that tilt and shift on these planes may impact the results. For the imaging SLM this is less of an issue because we have already marked the zero frequency and because a tilt of the imaging SLM only shifts the position of the spot on the sensor. However, if the illumination SLM is not registered correctly, we may see a sharp spot behind the

tissue, but it will be shifted from the bead of interest and will not excite it. Thus, we tilt and shift the modulation on the illumination SLM until the intensity we measure on the main camera (when the modulation correction is on) is maximized. After this is achieved we can fine-tune the shift on the imaging SLM, which is equivalent to the position of the zero (central) frequency that we have previously marked by looking at the illuminated circle.

To use a recovered modulation pattern to image a larger region of the fluorescent target, we leverage the tilt shift memory effect. To apply the scan we need to recover the parameter s of Eq. (21), determining the ratio between the tilt and shift. For that, after we recover the modulation pattern, we place it on the illumination SLM and use the validation camera to view the focused spot. We then adjust the ratio between tilt and shift of the modulation pattern so that we can move the focused spot in the validation camera, while preserving maximal intensity.

References

[1] Rotter, S. & Gigan, S. Light fields in complex media: Mesoscopic scattering meets wave control. *Rev. Mod. Phys.* **89**, 015005 (2017).

[2] Katz, O., Small, E., Guan, Y. & Silberberg, Y. Noninvasive nonlinear focusing and imaging through strongly scattering turbid layers. *Optica* **1**, 170–174 (2014).

[3] Ji, N. Adaptive optical fluorescence microscopy. *Nature Methods* **14**, 374–380 (2017).

[4] Boniface, A., Blochet, B., Dong, J. & Gigan, S. Noninvasive light focusing in scattering media using speckle variance optimization. *Optica* **6**, 1381–1385 (2019).

[5] Popoff, S. M. *et al.* Measuring the transmission matrix in optics: An approach to the study and control of light propagation in disordered media. *Physical Review Letters* **104**, 100601 (2010).

[6] Aizik, D., Gkioulekas, I. & Levin, A. Fluorescent wavefront shaping using incoherent iterative phase conjugation. *Optica* **9**, 746–754 (2022).

[7] Yeminy, T. & Katz, O. Guidestar-free image-guided wavefront shaping. *Science Advances* **7**, eabf5364 (2021).

[8] Osnabrugge, G., Horstmeyer, R., Papadopoulos, I. N., Judkewitz, B. & Vellekoop, I. M. Generalized optical memory effect. *Optica* **4**, 886–892 (2017).

[9] Bar, C., Alterman, M., Gkioulekas, L. & Levin, A. Single scattering modeling of speckle correlation. In *2021 IEEE International Conference on Computational Photography (ICCP)*, 1–16 (2021).

[10] Mugnier, L. M., Blanc, A. & Idier, J. Phase diversity: A technique for wave-front sensing and for diffraction-limited imaging. vol. 141 of *Advances in Imaging and Electron Physics*, 1–76 (Elsevier, 2006).
